# The Expression of Genes Involved in Phenylpropanoid Biosynthesis Correlates Positively with Phenolic Content and Antioxidant Capacity in Developing Chickpea (*Cicer arietinum* L.) Seeds

**DOI:** 10.3390/plants14162489

**Published:** 2025-08-11

**Authors:** Karen V. Pineda-Hidalgo, Gamaliel Flores-Paredes, José A. Garzón-Tiznado, Nancy Y. Salazar-Salas, Jeanett Chávez-Ontiveros, Gabriela López-Angulo, Francisco Delgado-Vargas, José A. Lopez-Valenzuela

**Affiliations:** Posgrado en Ciencia y Tecnología de Alimentos, Facultad de Ciencias Químico-Biológicas, Universidad Autónoma de Sinaloa, Culiacán 80013, Sinaloa, Mexico; kvpineda@uas.edu.mx (K.V.P.-H.); gamalielflores.fcqb@uas.edu.mx (G.F.-P.); garzon24@uas.edu.mx (J.A.G.-T.); nancy.salazar@uas.edu.mx (N.Y.S.-S.); jchontiveros@uas.edu.mx (J.C.-O.); gabylopez@uas.edu.mx (G.L.-A.); fdelgado@uas.edu.mx (F.D.-V.)

**Keywords:** chickpea, phenolics, antioxidants, MYB transcription factors, UPLC-MS

## Abstract

Chickpea (*Cicer arietinum* L.) seeds have a great diversity of phenolic compounds and antioxidant capacity, which is associated with the regulation of the phenylpropanoid pathway. We investigated this association in developing seeds (20 and 30 days after anthesis, DAA) from six chickpea genotypes (two kabuli and four desi). They were used to evaluate total phenolics (TP), total flavonoids (TF), phenolic composition, antioxidant capacity (AC), and the relative expression of MYB transcription factors (*CaMYB39*, *MYB111-like*, and *CaMYB92*) and phenylpropanoid biosynthetic genes (*PAL*, *CHI*, and *CHS*). TP, TF, and the AC increased significantly during seed development, and the highest values were observed in desi genotypes. The AC correlated with the levels of TP, TF, and the flavonols myricetin, quercetin, kaempferol, and isorhamnetin. The levels of the phenolic compounds and the AC also correlated positively with the expression of MYB transcription factors and phenylpropanoid biosynthetic genes. The expression of *CaMYB39* correlated significantly with that of *PAL*, *CHS*, and *CHI*, indicating the potential use of this MYB factor to improve the content of phenylpropanoids. The desi genotype with black seeds (ICC 4418) showed the highest levels of gene expression, TP, TF, and AC, suggesting it can be used to produce chickpeas with enhanced nutraceutical properties.

## 1. Introduction

Chickpea (*Cicer arietinum* L.) is the third most important food legume crop in the world and a rich source of compounds with nutritional and biological activities [1]. There is a great diversity of chickpea genotypes that show significant variations in seed quality traits (e.g., color, size, and protein content) and the content of phenolics and flavonoids [2,3], which have been associated with several biological activities, including antioxidant [4] and anticancer [5]. The genetic and biochemical diversity of chickpea can be used to improve the nutraceutical characteristics of commercial varieties.

The phenylpropanoid pathway produces metabolites crucial for plant survival and development, including bioactive compounds with beneficial effects on human health. Essential reactions for the biosynthesis of phenylpropanoids are catalyzed by phenylalanine ammonia-lyase (PAL), chalcone synthase (CHS), and chalcone isomerase (CHI). PAL catalyzes the conversion of phenylalanine to trans-cinnamic acid, a critical step for the entire pathway, while CHS and CHI are key enzymes for the synthesis of flavonoids. MYB transcription factors regulate the synthesis of phenylpropanoids, which represents an opportunity for plant breeders to improve the levels of bioactive compounds. Several R2R3-MYB proteins are involved in the control of flavonoid biosynthesis. In *Arabidopsis thaliana*, *At*MYB11, *At*MYB12, and *At*MYB111 are closely related and share the consensus motif ‘GRTxSRxMK’, which is unique to members of subgroup 7 (SG7) [6]. The three MYB factors promote the synthesis of flavonols by activating the transcription of *CHS*, *CHI*, flavanone 3-hydroxylase (*F3H*), and flavonol synthase 1 (*FLS1*); *At*MYB12 acts mainly in the root and *At*MYB111 in the cotyledons [7]. In *Cicer arietinum*, Rajput et al. [8] identified 119 MYB encoding genes that were grouped into 32 distinct functional clades based on phylogenetic analysis of the *Ca*MYB proteins with R2R3-MYBs from *A. thaliana* and other plant species. The authors demonstrated that two MYB factors of SG5, *Ca*PAR1 (*Ca*MYB89) and *Ca*PAR2 (*Ca*MYB98), regulate proanthocyanidin biosynthesis in chickpea seed coats. Singh et al. [9] analyzed two anthocyanin-specific SG6 R2R3-MYB transcription factors, *Ca*LAP1 (MYB90-like) and *Ca*LAP2 (MYB114-like), whose overexpression increased the accumulation of anthocyanins and proanthocyanidins in chickpea seed coats. Saxena et al. [10] characterized a MYB11-like transcription factor named *Ca*MYB39 (SG7) and found that it regulates the synthesis of flavonols mainly in chickpea trichomes and, to a lesser extent, in other tissues like developing seeds. *Ca*MYB39 activated the transcription of *CHS*, *CHI*, *F3H*, *F3’H*, and *FLS* to induce flavonol biosynthesis, and its overexpression conferred tolerance to ascochyta blight. These results demonstrate that MYB transcription factors with the amino acid motifs of subgroups 5 to 7 regulate the phenylpropanoid pathway in chickpea, suggesting they are good candidates for the genetic manipulation of phenylpropanoid biosynthesis to improve the nutritional value and disease resistance of this legume.

Quintero-Soto et al. [4] previously demonstrated a significant variability in the content of phenolic compounds and antioxidant capacity in the mature seeds of some kabuli chickpeas from Mexico and some desi genotypes from other countries. They identified some promising kabuli genotypes (Blanco Sinaloa 92 and Blanoro) with high levels of phenolic acids and some desi genotypes (ICC 4418 and 6306) with remarkably high levels of flavonoids, suggesting the up-regulation of key genes from the phenylpropanoid pathway in these genotypes. This research aimed to analyze the transcript levels of some MYB transcription factors and phenylpropanoid biosynthetic genes, as well as their relationship with the content of phenolic compounds and the antioxidant capacity in developing seeds of selected chickpea genotypes.

## 2. Results and Discussion

### 2.1. Antioxidant Capacity (AC)

The AC registered in the methanol extracts varied considerably among the six chickpea genotypes (Table 1). The desi genotypes showed the highest AC for the three assays, and their average values were 3–8 times higher than those of the kabuli genotypes. The desi chickpea with black seeds (ICC 4418) showed the highest AC value at 30 DAA. The AC increased significantly in almost all genotypes during seed development, reaching values at 30 DAA similar to those reported by Quintero-Soto et al. [4] in mature seeds of the same genotypes. These results indicate that the chickpea genotypes represent good sources of antioxidants. This is relevant considering that people in Mexico and other countries consume roasted or steamed green chickpeas as a snack.

### 2.2. Phenolic and Flavonoid Content and Their Association with the AC

**Figure 1:**
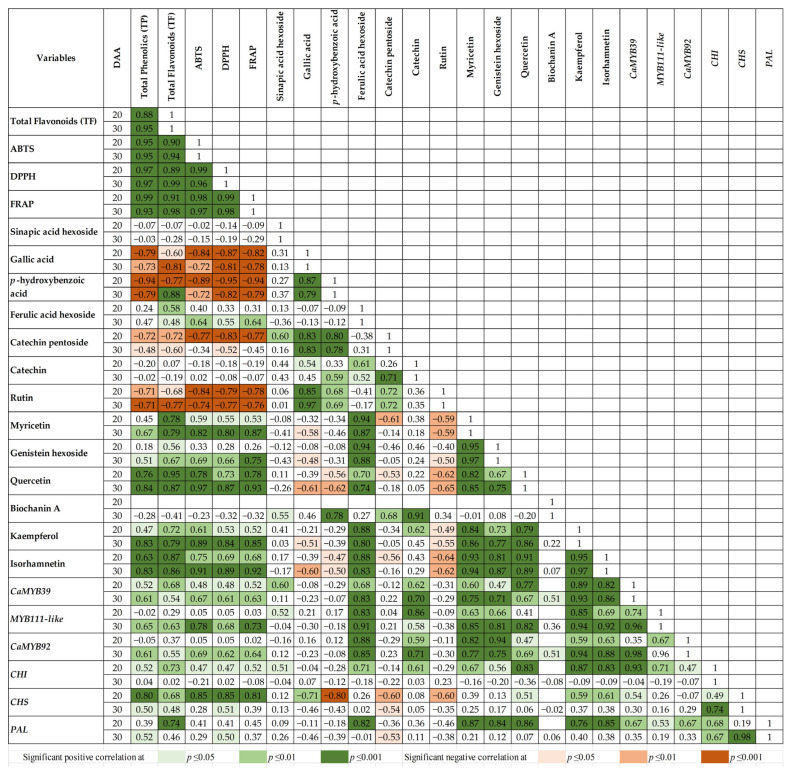
Pearson correlation coefficients between phenolic content, antioxidant capacity, and gene expression levels of chickpea genotypes. Only compounds that were detected in at least 50% of the genotypes were included.

The total phenolics (TP) content of the methanol extracts (22.2–263.6 mg GAE/100 g) (Table 1) varied significantly among the chickpea genotypes. The TP increased significantly from 20 to 30 DAA, with the highest increase observed in the black chickpea ICC 4418. The values corresponded with those reported previously in mature seeds of the same genotypes [2]. The variation in TP showed strong correlations with the AC evaluated by the three assays: ABTS (r = 0.95; *p* ≤ 0.001), DPPH (r = 0.97; *p* ≤ 0.001), and FRAP (r = 0.93–0.99; *p* ≤ 0.001) at both developmental stages (Figure 1).

The total flavonoids (TF) content at 20 and 30 DAA (28.5–220.2 mg CAE/100 g) also showed strong correlations with the AC by the three assays: ABTS (r = 0.90–0.94; *p* ≤ 0.001), DPPH (r = 0.89–0.99; *p* ≤ 0.001), and FRAP (r = 0.91–0.98; *p* ≤ 0.001) (Figure 1). Like TP, the TF content increased during seed development. The genotype with black seeds (ICC 4418) showed the highest value at 30 DAA, being 6.3-fold higher than that of Blanco Sinaloa 92 (34.9 mg CAE/100 g). ICC 4418 also showed the highest content of TF in mature seeds [4].

**Figure 2:**
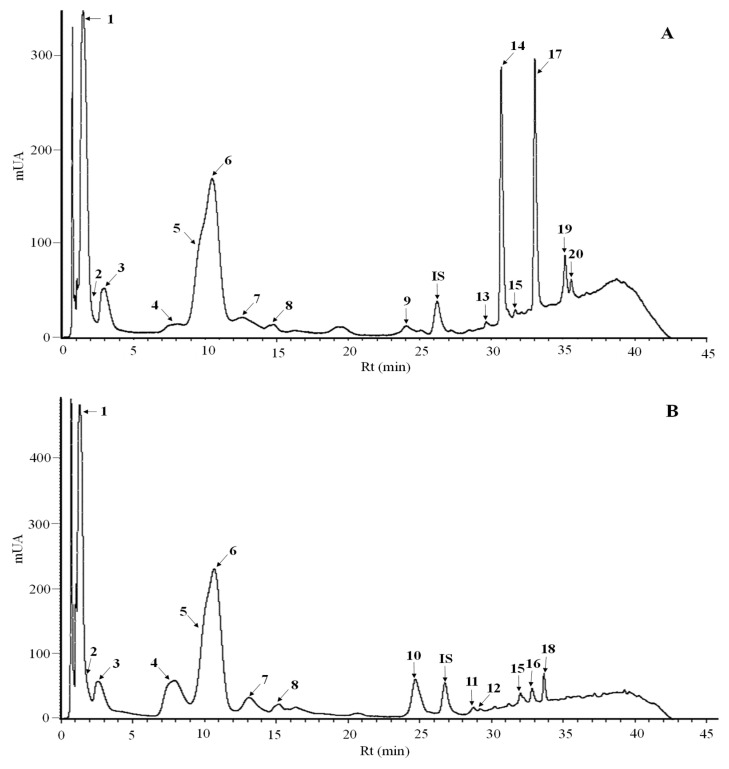
UPLC-DAD chromatographic separation of methanol extracts from developing seeds of the chickpea genotypes ICC 4418 (**A**) and Blanco Sinaloa 92 (**B**). Peaks were identified as sinapic acid hexoside (1), gallic acid (2), catechin pentoside (3), dihidroxybenzoic acid (4), catechin (5), vanillic acid (6), *p*-hydroxybenzoic acid (7), benzoic acid (8), *p*-coumaric acid (9), ferulic acid hexoside (10), myricetin-*O*-methyl ether hexoside deoxyhexoside pentoside (11), myricetin-*O*-methyl ether hexoside deoxyhexoside (12), rutin (13), myricetin (14), genistein hexoside (15), isorhamnetin 3-*O*-*β*-D-glucopyranoside (16), quercetin (17), biochanin A (18), kaempferol (19), and isorhamnetin (20). The 2-hydrocinnamic acid was used as an internal standard (IS).

The UPLC analysis of the chickpea methanol extracts separated 20 metabolites (Figure 2) that were identified by mass spectrometry (Table S1). The most abundant phenolics (mg/kg dw) were sinapic acid hexoside (14.2–171.6) and gallic acid (10.4–215.5) (Table 2), as well as the flavonoids catechin pentoside (23.7–90), catechin (8.9–63.1), and isorhamnetin (Nd-51.2) (Table 3). The levels of gallic acid, *p*-hydroxybenzoic acid, and catechin pentoside were significantly higher in kabuli than desi genotypes (Table 2), while the opposite was observed for the flavonols myricetin, quercetin, kaempferol, and isorhamnetin (Table 3). These results agree with previous studies in developing and mature seeds that showed significantly higher levels of flavonols in desi than kabuli chickpeas [4,11,12]. The chickpea with black seeds (ICC 4418) showed the highest levels of most flavonoids. The levels of myricetin, quercetin, kaempferol, and isorhamnetin showed positive correlations with the AC by the three methods at both developmental stages (Figure 1).

### 2.3. Expression of MYB Transcription Factors and Target Genes

**Figure 3:**
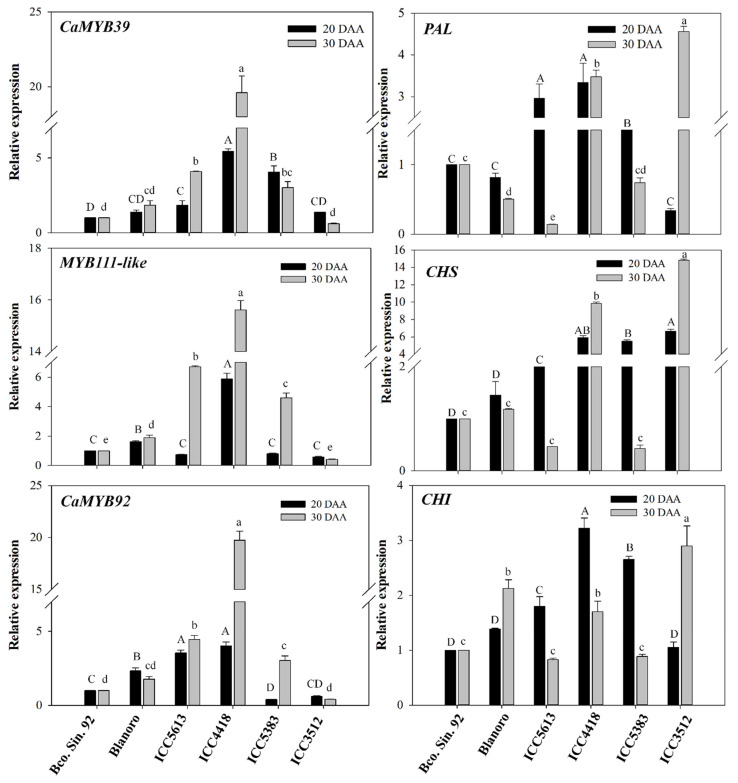
Expression of MYB transcription factors and phenolic biosynthetic genes in developing seeds of chickpea. The *eEF1A* gene was used as control, and the transcript levels were calculated relative to those of the genotype Blanco Sinaloa 92. DAA, days after anthesis. Different capital and lowercase letters indicate significant differences among the genotypes at 20 DAA and 30 DAA, respectively (Fisher, *p* < 0.05). MYB transcription factors: *CaMYB39*, *MYB111-like*, and *CaMYB92*. Key phenolic biosynthetic genes: *PAL*, phenylalanine ammonia-lyase; *CHS*, chalcone synthase; *CHI*, chalcone isomerase.

To investigate if the high content of phenolic compounds in the desi chickpea genotypes is associated with the up-regulation of genes from the phenylpropanoid pathway, the expression of MYB transcription factors [*CaMYB39* (MYB11-like, SG7), *MYB111-like* (SG7), and *CaMYB92* (MYB114-like, SG5)] and some phenylpropanoid biosynthetic genes [*PAL*, *CHS*, and *CHI*] was evaluated (Figure 3). The middle (20 DAA) and late stages (30 DAA) of seed development were chosen because a transcriptome analysis of chickpea suggested that flavonoid biosynthesis is closely associated with seed maturation [13]. The expression of *CaMYB39* varied significantly among the chickpea genotypes; the transcript level of the genotype with black seeds (ICC 4418) was about 20 times higher than that of Blanco Sinaloa 92 at 30 DAA, which corresponded with the highest phenolic and flavonoid content registered in ICC 4418 (Table 1). CaMYB39 contains the consensus motif ‘GRTxSRxMK’ unique to members of SG7 [6] and regulates flavonol biosynthesis in chickpea [10]. This agrees with the correlation registered in the present research between the transcript levels of *CaMYB39* and the content of the flavonols myricetin (r = 0.60–0.75; *p* ≤ 0.01), quercetin (r = 0.67–0.77; *p* ≤ 0.01), kaempferol (r = 0.89–0.93; *p* ≤ 0.01), and isorhamnetin (r = 0.82–0.86; *p* ≤ 0.01) at 20 and 30 DAA. The expression of *CaMYB39* also correlated with the AC measured by the three assays (r = 0.48–0.67; *p* ≤ 0.05), total phenolics (r = 0.52–0.61; *p* ≤ 0.05), total flavonoids (r = 0.54–0.68; *p* ≤ 0.05), ferulic acid hexoside (r = 0.68–0.83; *p* ≤ 0.01), catechin (r = 0.62–0.70; *p* ≤ 0.01), and genistein hexoside (r = 0.47–0.71; *p* ≤ 0.05) (Figure 1). These results suggest the potential use of *CaMYB39* as a marker to improve the content of flavonoids in chickpea.

*MYB111-like* also contains the consensus motif unique to SG7 members that promotes the synthesis of flavonols [6]. The expression pattern of this transcription factor at 30 DAA (Figure 3) showed significant correlations with the AC by the three assays (r = 0.68–0.78; *p* ≤ 0.01) and with the levels of ferulic acid hexoside (r = 0.91; *p* ≤ 0.001), catechin (r = 0.58; *p* ≤ 0.05), genistein hexoside (r = 0.81; *p* ≤ 0.001), and the flavonols myricetin (r = 0.85; *p* ≤ 0.001), quercetin (r = 0.82; *p* ≤ 0.001), kaempferol (r = 0.94; *p* ≤ 0.001), and isorhamnetin (r = 0.92; *p* ≤ 0.001) (Figure 1).

*CaMYB92* was identified by Rajput et al. [8] as a candidate proanthocyanidin regulator (SG5) that is expressed in developing chickpea seeds. The expression pattern of this gene was like that of the other MYB factors at 30 DAA (Figure 3). It also showed similar correlations with the AC, total phenolics, total flavonoids, as well as with the compounds ferulic acid hexoside, catechin, genistein hexoside, and the flavonols myricetin, quercetin, kaempferol, and isorhamnetin (Figure 1).

PAL, CHI, and CHS catalyze crucial reactions in the phenylpropanoid pathway, and the expression of their genes is regulated by MYB transcription factors. The transcript levels of *PAL* varied considerably among the chickpea genotypes (Figure 3) and, at 20 DAA, correlated with the expression of *CaMYB39* (r = 0.67; *p* ≤ 0.01), *MYB111-like* (r = 0.53; *p* ≤ 0.05), and *CaMYB92* (r = 0.67; *p* ≤ 0.01). The transcript levels of *PAL* at 20 DAA also correlated significantly with total flavonoids (r = 0.74; *p* ≤ 0.001) and the content of ferulic acid hexoside (r = 0.82; *p* ≤ 0.001), genistein hexoside (r = 0.84; *p* ≤ 0.001), myricetin (r = 0.87; *p* ≤ 0.001), quercetin (r = 0.86; *p* ≤ 0.01), kaempferol (r = 0.76; *p* ≤ 0.001), and isorhamnetin (0.85; *p* ≤ 0.001) (Figure 1).

CHS catalyzes the first step in flavonoid biosynthesis and therefore is essential for this pathway. The expression of the gene encoding this enzyme in chickpea is activated by *Ca*MYB39 [10]. In this regard, the expression of *CHS* in the chickpea genotypes correlated with the transcript levels of *CaMYB39* at 20 DAA (Figure 1 and Figure 3). The expression of *CHS* at 20 DAA also showed significant correlations with total phenolics (r = 0.80; *p* ≤ 0.001), total flavonoids (r = 0.68; *p* ≤ 0.01), AC (r = 0.81–0.85; *p* ≤ 0.001), and the flavonols quercetin (r = 0.51; *p* ≤ 0.05), kaempferol (r = 0.59; *p* ≤ 0.01), and isorhamnetin (0.61; *p* ≤ 0.01) (Figure 1), supporting the important role of this enzyme in the synthesis of flavonoids.

CHI is another key enzyme in flavonoid biosynthesis that converts naringenin chalcone to naringenin. Its expression in tomato seems to be regulated by *SlMYB12*, another member of SG7 [14]. The expression of *CHI* varied significantly among the chickpea genotypes (Figure 3) and at 20 DAA showed a high correlation with *CaMYB39* (r = 0.93; *p* ≤ 0.001) and *MYB111-like* (r = 0.71; *p* ≤ 0.01), which share the consensus motif of SG7. The transcript levels of this gene at 20 DAA also correlated with the content of total flavonoids (r = 0.73; *p* ≤ 0.01), ferulic acid hexoside (r = 0.71; *p* ≤ 0.001), catechin (r = 0.61; *p* ≤ 0.01), genistein hexoside (r = 0.56; *p* ≤ 0.05), and the flavonols myricetin (r = 0.67; *p* ≤ 0.01), quercetin (r = 0.83; *p* ≤ 0.001), kaempferol (r = 0.87; *p* ≤ 0.001), and isorhamnetin (0.83; *p* ≤ 0.001) (Figure 1).

### 2.4. Associations Between Phenolic Content, Antioxidant Capacity, and Gene Expression

**Figure 4:**
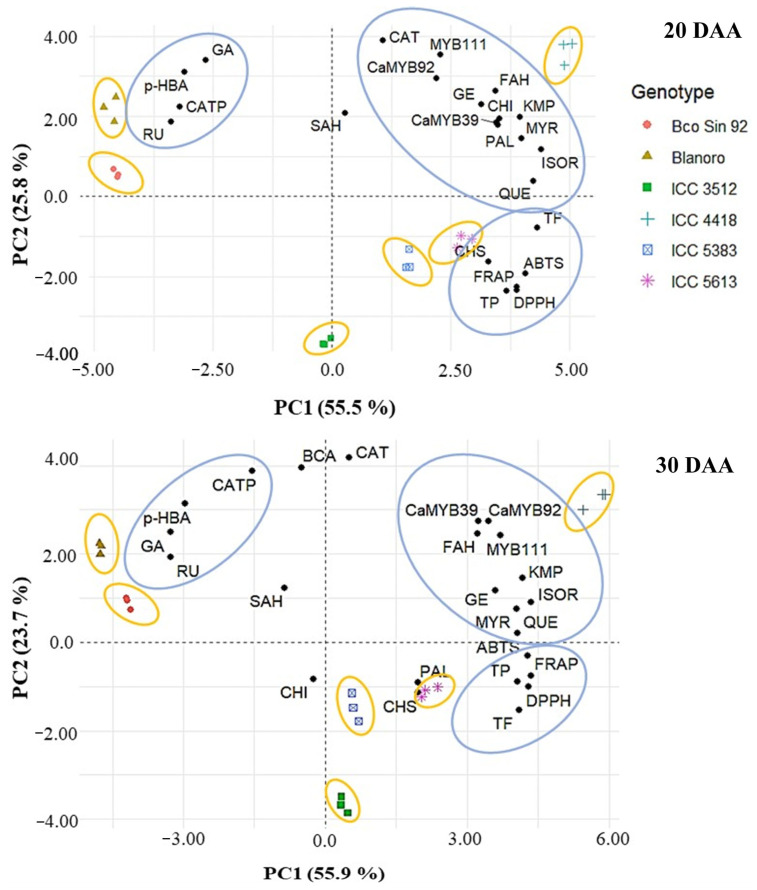
Biplots based on principal component analysis of phenolics and flavonoids content, antioxidant capacity, and gene expression of developing seeds (20 and 30 DAA) from six chickpea genotypes. Genotypes are depicted by colored shapes and the variables by black points. FAH: ferulic acid hexoside; KMP: kaempferol; ISOR: isorhamnetin; GE: genistein hexoside; MYR: myricetin; QUE: quercetin; CAT: catechin; CATP: catechin pentoside; *p*-HBA: *p*-Hydroxybenzoic acid; GA: gallic acid; RU: rutin; SAH: sinapic acid hexoside; TP: total phenolics; TF: total flavonoids; antioxidant capacity: ABTS, DPPH, and FRAP. MYB transcription factors: *CaMYB39*, *MYB111*, and *CaMYB92*. Key phenolic biosynthetic genes: *PAL*, phenylalanine ammonia-lyase; *CHS*, chalcone synthase; *CHI*, chalcone isomerase.

Principal component analysis (PCA) of phenolic and flavonoid content, antioxidant capacity, and gene expression of the six chickpea genotypes explained 81.3% and 79.6% of the variation at 20 DAA and 30 DAA, respectively (Figure 4). Three groups were formed between the variables at both developmental stages. At 20 DAA, the group in the upper right quadrant was formed by flavonoids (mainly flavonols), MYB transcription factors, *PAL*, and *CHI* and was very close to the desi genotype ICC 4418 (black). The group in the lower right quadrant was formed by TP, TF, AC, and *CHS*, being very close to the desi genotype ICC 5613 (green). At 30 DAA, the group in the upper right quadrant was formed by the same metabolites and MYB factors, but *PAL* and *CHI* were colocalized with the desi genotype ICC 5613 (green). The third group in the upper left quadrant was formed by two phenolic acids (gallic and *p*-hydroxybenzoic) and two flavonoids (catechin pentoside and rutin). These metabolites were more abundant in the kabuli genotypes, and consequently, Blanco Sinaloa 92 and Blanoro were located close to this group at both developmental stages. This analysis highlighted that ICC 4418 showed the highest levels of metabolites and expression of MYB transcription factors and key genes involved in phenolic biosynthesis.

## 3. Materials and Methods

### 3.1. Plant Material

Six chickpea (*Cicer arietinum* L.) genotypes were used: four desi (ICC3512, 4418, 5383, and 5613) from ICRISAT and two kabuli (Blanco Sinaloa 92 and Blanoro) from INIFAP (National Institute of Forestry, Agriculture and Livestock Research). The genotypes were grown during the winter–spring season of 2016–2017 at the experimental field of INIFAP in Culiacan, Sinaloa, Mexico (24°36′58″ N, 107°25′48″ W), as described by Chavez-Ontiveros et al. [2]. A randomized complete block design with three replications was used. Developing seeds from each replicate were obtained 20 and 30 days after anthesis (DAA), frozen in liquid nitrogen, and then stored at −80 °C until use. For metabolite analysis, the seeds were lyophilized and then ground (Retsch MM400, Haan, Germany) to pass a 60-mesh sieve.

### 3.2. Preparation of Methanol Extracts

Methanol extracts were obtained as described by Quintero-Soto et al. [4]. Dry chickpea flour (0.75 g) from each replicate was mixed with 30 mL of 80% methanol. The mixture was agitated at 300 rpm for 30 min, sonicated for 20 min, and then hydrolyzed for 30 min at 90 °C with 12 mL of 2 mol/L HCl. The supernatant recovered after centrifugation (15,000× *g*, 20 min) was extracted with hexane (30 mL), and the aqueous phase was mixed with 50 mL of water and 30 mL of ethyl acetate. The recovered fraction in ethyl acetate was concentrated with a rotary evaporator (BÜCHI R-124, Brinkmann Instruments, Westbury, NY, USA). The extract was dissolved in 80% methanol (0.75 mL), passed through a PVDF filter (0.45 μm, Pall, Port Washington, NY, USA), and stored at −20 °C until use.

### 3.3. Total Phenolics (TP) and Flavonoids (TF)

Methanol extracts (equivalent to 1 mg of dry flour/µL) were used to measure TP by the Folin–Ciocalteu reaction [15] and TF [16]. For TP, 20 µL of the extract or methanol (blank) was mixed in a microplate with 220 µL of Folin–Ciocalteu reagent (diluted 8 times), stirred for 3 min, and the mixture was added with 60 µL of 7% sodium carbonate solution. The sample was incubated in darkness (90 min at 25 °C), and the absorbance was measured at 765 nm (Multiskan Sky, Thermo Fisher, Waltham, MA, USA). Gallic acid was dissolved in methanol (1 mg/mL) and diluted to prepare a standard curve (0.0078125–0.5 mg/mL). TP content was expressed as milligrams of gallic acid equivalents (GAEs) per 100 g of dry weight (dw). For TF, 25 µL of the extract or methanol (blank) was mixed with 120 µL of deionized water and 6 µL of a 5% NaNO_2_ solution. After 5 min, the mixture was added with 12 µL of 10% AlCl_3_, incubated for 5 min, and then added with 60 µL of 1 mol/L NaOH. The absorbance was registered at 510 nm (Multiskan Sky, Thermo Fisher, Waltham, MA, USA). Catechin was used to prepare a standard curve (0.025–0.2 mg/mL), and the results were reported as catechin equivalents (CAEs) per 100 g of dry weight (dw).

### 3.4. Phenolic Profiles

Phenolic profiles were obtained as indicated by Quintero-Soto et al. [4] using a UPLC-DAD system (ACCELA, Thermo Scientific, Waltham, MA, USA). The methanol extract (5 µL) was separated with a C18 column (Fortis, 3 µm, 50 × 2.1 mm) (Fortis Technologies Ltd., Neston, UK) using formic acid (1%, *v*/*v*) (A) and acetonitrile (B) as mobile phases. The linear gradient used was 0.5–60% B during 40 min, and the flow rate was 0.2 mL/min. The compounds were detected at 280, 320, and 350 nm. The internal standard was 2-hydroxycinnamic acid (10 µg/mL). The compounds were quantified using the following calibration curves: phenolic acids (gallic, *p*-coumaric, *p*-hydroxybenzoic, ferulic, sinapic, and vanillic) and flavonoids (biochanin A, catechin, genistein, isorhamnetin, kaempferol, myricetin, quercetin, and rutin) (Sigma-Aldrich, St. Louis, MO, USA). The phenolic content was reported in micrograms per gram of dry weight (µg/g dw). The UPLC-DAD system was connected to a linear ion trap mass spectrometer (MS) equipped with an ESI source (LTQ XL, Thermo Scientific, Waltham, MA, USA). The spectra (*m*/*z* = 50–1500) were acquired in negative/positive mode (35 V, 300 °C). Helium was used for collision-induced dissociation (10–45 V) in MS^n^ experiments, and nitrogen was used for drying. The Xcalibur 2.2 software (Thermo Scientific, Waltham, MA, USA) was used for data analysis.

### 3.5. Antioxidant Capacity (AC)

The AC of the methanol extracts (equivalent to 1 mg of dry flour/µL) was analyzed based on the ABTS (2,2′-Azino-bis (3-ethylbenzothiazoline-6-sulfonic acid) [17], DPPH (1,1-diphenyl-2-picrylhydrazyl) [18], and FRAP (Ferric Reducing Antioxidant Power) [19] methods. The assays were carried out using 96-well microplates. For ABTS, 7.5 µL of the extract or methanol (blank) was mixed with 192.5 µL of the radical solution (Abs 734 nm = 0.7). The mixture was allowed to react for 10 min at 25 °C in darkness, and then, the absorbance was measured at 734 nm (Multiskan Sky, Thermo Fisher, Waltham, MA, USA). For DPPH, the radical was dissolved in methanol (150 µmol/L), and 180 µL was mixed with 20 µL of each extract or methanol (blank). The sample was incubated for 30 min at 37 °C, and the absorbance was measured at 510 nm (Multiskan Sky, Thermo Fisher, Waltham, MA, USA). For FRAP, the reagent was prepared with 10 mL of 10 mmol/L TPTZ (2,4,6-tripyridyl-s-triazine) in 40 mmol/L HCl, 10 mL of 20 mmol/L FeCl_3_•6H_2_O, and 100 mL of 300 mmol/L sodium acetate buffer, pH 3.6. The methanol extract (20 µL) or methanol (blank) was mixed with 280 µL of the FRAP reagent and incubated in darkness for 30 min at room temperature. The absorbance was measured at 595 nm (Multiskan Sky, Thermo Fisher, Waltham, MA, USA). For the AC quantitation, the three assays used a Trolox standard curve (0.05–1.0 µmol/mL), and the results were reported as micromoles of Trolox Equivalents per 100 g of dry weight (µmol TE/100 g dw).

### 3.6. RNA Isolation

Total RNA was extracted from developing seeds (20 and 30 DAA) as described by Holding et al. [20] with some modifications. About 0.6 g of seeds were ground in liquid nitrogen, washed with 90% acetone and then centrifuged (15,000× *g*, 15 min, 4 °C). Approximately 0.1 g of the pellet was homogenized with 200 µL of RNA extraction buffer [50 mmol/L Tris-HCl, pH 8, 150 mmol/L LiCl, 5 mmol/L EDTA, pH 8, and 10 g/L SDS]. The sample was mixed with 200 µL of phenol/chloroform (1:1) at pH 8.0, incubated on ice for 5 min, and then centrifuged (10,000× *g*, 10 min, 4 °C) to recover the aqueous phase. To remove the starch, the sample was added with 1 mL of trizol reagent (Life Technologies, Carlsbad, CA, USA) and then with 200 µL of phenol–chloroform (1:1). After centrifugation (10,000× *g*, 10 min, and 4 °C), the RNA was precipitated from the aqueous phase with one volume of isopropanol. The RNA pellet recovered after centrifugation (10,000× *g*, 10 min, and 4 °C) was washed with 70% ethanol, dried, and resuspended in 50 µL of DEPC water. The sample was treated with LiCl and sodium acetate. The Kit DNAse I (Sigma-Aldrich, St. Louis, MO, USA) was used to remove any contaminant DNA.

### 3.7. Gene Expression Analysis

RT-qPCR was used to analyze the expression of MYB transcription factors (*CaMYB39*, *MYB111-like*, and *CaMYB92*) and phenylpropanoid biosynthetic genes (*PAL*, *CHS*, and *CHI*). For the reactions, about 20 ng of RNA were used with the SCRIPT One-Step RT-PCR kit (Jena Bioscience, Jena, Germany). Primer-BLAST http://www.ncbi.nlm.nih.gov/tools/primer-blast (accessed on 21 May 2023) was used to obtain the PCR primer sequences (Table S2). The reactions were performed using the StepOnePlusTM real-time system (Applied Biosystems, Carlsbad, CA, USA) with the following conditions: initial period for cDNA synthesis at 50 °C for 30 min, followed by denaturation at 95 °C for 10 min, then 40 cycles at 95 °C (15 s) and 60 °C (1 min), and gradient of 2.2 °C/s. The relative expression values were calculated using the 2^–ΔΔCt^ method [21]. Elongation factor 1A (eEF1A) was used as a control gene, and the genotype Blanco Sinaloa 92 was used as the reference sample. Bar graphs of relative gene expression were constructed using SigmaPlot 12.0 (SYSTAT Software Inc., San Jose, CA, USA).

### 3.8. Statistical Analysis

Data from three replicates were analyzed by two-way ANOVA. The Fisher test (α = 0.05) was used to compare the means among the chickpea genotypes and between the developmental stages. Pearson’s correlation analysis was also carried out using the software STATGRAPHICS Plus version 5.1 (Statistical Graphics Corporation™, Warrenton, VA, USA). A principal component analysis (PCA) was conducted to establish the relationship between metabolites and the antioxidant capacity of the genotypes using RStudio version 2024.12.1 (R Foundation for Statistical Computing, Vienna, Austria). FactoMineR and factoextra packages were used for PCA analysis and visualization, respectively.

## 4. Conclusions

The content of phenolics and the AC of the chickpea genotypes increased significantly during seed development. The highest AC values were observed in desi genotypes and were mainly associated with the content of flavonols. The levels of the phenolic compounds and the AC of the chickpea genotypes correlated positively with the relative expression of Ca*MYB39*, *MYB111-like*, and *CaMYB92* and the phenylpropanoid biosynthetic genes *PAL*, *CHI*, and *CHS*. Thus, MYB factors could be used as markers in breeding programs to produce chickpeas with better nutraceutical properties. The genotype ICC 4418 showed the highest relative gene expression, phenolic compounds content, and AC, suggesting it can be used to improve the levels of phenylpropanoids and the antioxidant capacity.

## Figures and Tables

**Table 1:** Total phenolics, total flavonoids, and antioxidant capacity of methanol extracts from developing seeds of chickpea genotypes.

Chickpea Genotype	Seed Color	Phenolics Content (mg GAE/100 g)		Flavonoids Content (mg CAE/100 g)		Antioxidant Capacity (µmol TE/100 g)					
						ABTS		DPPH		FRAP	
		20 DAA	30 DAA	20 DAA	30 DAA	20 DAA	30 DAA	20 DAA	30 DAA	20 DDA	30 DDA
Kabuli											
Bco Sin 92	Cream	24.1 ± 0.9 ^Db^	45.1 ± 4.4 ^Ea^	28.5 ± 0.8 ^Ca^	34.9 ± 0.7 ^Da^	389.6 ± 10.4 ^Ba^	421.1 ± 3.4 ^Ea^	145.1 ± 6.5 ^Ca^	186.6 ± 2.2 ^Ca^	86.60 ± 1.2 ^Ea^	102.8 ± 3.6 ^Ea^
Blanoro	Cream	22.2 ± 2.2 ^Db^	39.4 ± 0.6 ^Ea^	43.6 ± 2.8 ^Ca^	37.6 ± 1.9 ^Da^	240.7 ± 20.9 ^Cb^	311.8 ± 2.1 ^Fa^	106.8 ± 6.3 ^Ca^	125.2 ± 4.5 ^Da^	40.00 ± 6.7 ^Fb^	90.9 ± 4.9 ^Ea^
Desi											
ICC 5613	Green	147.6 ± 7.7 ^Ba^	154.7 ± 1.5 ^Da^	152.4 ± 6.6 ^Ab^	187.6 ± 7.9 ^Ba^	900.8 ± 31.4 ^Ab^	1366.4 ± 41.1 ^Ca^	736.8 ± 6.9 ^Ab^	1157.4 ± 55.8 ^Ba^	545.9 ± 18.3 ^Bb^	856.6 ± 7.8 ^Ba^
ICC 4418	Black	124.4 ± 11.5 ^Cb^	263.6 ± 3.9 ^Aa^	147.9 ± 6.8 ^Ab^	220.2 ± 9.9 ^Aa^	917.7 ± 11.4 ^Ab^	1794.9 ± 63.8 ^Aa^	659.3 ± 21.8 ^Bb^	1590.6 ± 26.8 ^Aa^	467.8 ± 0.9 ^Db^	1014.5 ± 17.5 ^Aa^
ICC 5383	Brown	187.9 ± 1.7 ^Ab^	226.9 ± 7.5 ^Ba^	144.9 ± 7.2 ^Ab^	177.1 ± 7.8 ^Ca^	959.1 ± 15.7 ^Ab^	1490.1 ± 17.2 ^Ba^	765.3 ± 0.9 ^Ab^	1171.1 ± 9.5 ^Ba^	622.3 ± 17.2 ^Ab^	729.1 ± 2.4 ^Ca^
ICC 3512	Brown	143.6 ± 1.1 ^Bb^	193.1 ± 1.3 ^Ca^	94.6 ± 7.0 ^Bb^	180.9 ± 2.1 ^Ba^	911.4 ± 17.4 ^Ab^	1003.8 ± 11.1 ^Da^	771.2 ± 2.9 ^Ab^	1175.7 ± 7.4 ^Ba^	516.1 ± 7.1 ^Cb^	652.3 ± 36.6 ^Da^

Values expressed on a dry weight basis. Mean ± standard deviation (three replicates). GAEs, gallic acid equivalents; CAEs, catechin equivalents; DAA, days after anthesis. For the same parameter, different superscript letters in the same column (A, B, C, D, E and F) or row (a and b) indicate significant differences (Fisher test, *p* ≤ 0.05) among genotypes and between developmental stages, respectively.

**Table 2:** Phenolic acid content of methanol extracts from developing seeds of chickpea genotypes.

Metabolite	DAA	Genotype						
		Bco. Sin. 92	Blanoro	ICC 5613	ICC 4418	ICC 5383	ICC 3512	LOD
Sinapic acid hexoside ^z^	20	124.89 ± 13.50 ^Ab^	70.50 ± 5.82 ^Bb^	14.20 ± 1.22 ^Ca^	143.71 ± 15.89 ^Aa^	132.37 ± 13.95 ^Aa^	56.30 ± 5.69 ^Bb^	0.14
	30	171.62 ± 19.94 ^Aa^	92.72 ± 6.22 ^Ca^	32.78 ± 3.73 ^Da^	135.29 ± 16.69 ^Ba^	130.48 ± 15.77 ^Ba^	107.38 ± 10.18 ^Ca^	
Gallic acid	20	113.25 ± 8.86 ^Ba^	169.28 ± 4.71 ^Ab^	44.66 ± 2.69 ^Ea^	87.88 ± 7.62 ^Ca^	69.11 ± 9.27 ^Da^	10.42 ± 1.83 ^Fb^	0.14
	30	108.96 ± 13.88 ^Ba^	215.52 ± 5.33 ^Aa^	39.20 ± 0.69^Da^	58.55 ± 6.23 ^Cb^	70.03 ± 9.33 ^Ca^	29.68 ± 2.62 ^Da^	
Dihydroxybenzoic acid	20	<LOD	5.61 ± 0.14 ^b^	<LOD	<LOD	<LOD	<LOD	0.05
	30	<LOD	13.41 ± 0.38 ^a^	<LOD	<LOD	<LOD	<LOD	
Vanillic acid	20	<LOD	<LOD	1.79 ± 0.13 ^Ab^	0.68 ± 0.21 ^Bb^	<LOD	<LOD	0.04
	30	<LOD	<LOD	6.79±1.01 ^Aa^	2.47 ±0.31 ^Ba^	<LOD	<LOD	
*p*-Hydroxybenzoic acid	20	41.60 ± 3.15 ^Ab^	38.91 ± 5.92 ^Ab^	11.58 ± 0.47 ^Ca^	22.79 ± 2.64 ^Ba^	8.59 ± 0.26 ^Ca^	5.23 ± 0.04 ^Da^	0.05
	30	48.88 ± 3.64 ^Aa^	45.73 ± 2.51 ^Aa^	13.20 ± 0.29 ^Ca^	24.02 ± 0.86 ^Ba^	10.27 ± 0.08 ^Ca^	5.26 ± 0.03 ^Da^	
Benzoic acid	20	<LOD	6.44 ± 0.44 ^Bb^	<LOD	9.48 ± 0.50 ^Ab^	<LOD	<LOD	0.05
	30	<LOD	7.35 ± 0.16 ^Ba^	<LOD	10.66 ± 0.28 ^Aa^	<LOD	<LOD	
*p*-Coumaric acid	20	<LOD	<LOD	<LOD	<LOD	<LOD	<LOD	0.12
	30	<LOD	34.06 ± 0.37	<LOD	<LOD	<LOD	<LOD	
Ferulic acid hexoside ^z^	20	0.58 ± 0.07 ^Ca^	1.26 ± 0.04 ^Cb^	5.19 ± 0.95 ^Ba^	9.16 ± 1.10 ^Aa^	0.95 ± 0.11 ^Cb^	0.49 ± 0.04 ^Ca^	0.11
	30	1.02 ± 0.03 ^Ea^	3.71 ± 0.34 ^Ca^	5.90 ± 0.69 ^Ba^	7.96 ± 0.23 ^Ab^	2.77 ± 0.02 ^Da^	1.26 ± 0.01 ^Ea^	

Values expressed in µg/g on a dry weight basis. Mean ± standard deviation (three replicates). ^z^ µg aglycone equivalent/g of sample; DAA, days after anthesis. For the same compound, different capital letters in the same row (A, B, C, D, E and F) or lowercase letters in the same column (a and b) show significant differences (Fisher test, *p* < 0.05) among genotypes and between developmental stages, respectively. LOD: limit of detection (µg/mL).

**Table 3:** Flavonoid content of methanol extracts from developing seeds of chickpea genotypes.

Metabolite	DAA	Genotype						
		Bco. Sin. 92	Blanoro	ICC 5613	ICC 4418	ICC 5383	ICC 3512	LOD
Catechin pentoside ^z^	20	77.88 ± 6.79 ^Aa^	72.95 ± 3.88 ^Ab^	23.74 ± 1.47 ^Eb^	47.82 ± 1.94 ^Cb^	57.36 ± 4.58 ^Ba^	31.86 ± 3.64 ^Da^	0.09
	30	69.58 ± 2.74 ^Bb^	89.99 ± 5.03 ^Aa^	58.66 ± 2.09 ^Ca^	71.88 ± 0.61 ^Ba^	60.60 ± 6.62 ^Ca^	38.52 ± 4.06 ^Da^	
Catechin	20	9.93 ± 0.82 ^Db^	42.64 ± 3.27 ^Ba^	9.85 ± 1.02 ^Da^	56.89 ± 3.76 ^Ab^	18.69 ± 2.23 ^Ca^	8.87 ± 0.93 ^Da^	0.09
	30	35.54 ± 2.49 ^Ca^	44.69 ± 4.22 ^Ba^	12.72 ± 1.32 ^Ea^	63.13 ± 5.17 ^Aa^	19.14 ± 0.36 ^Da^	11.17 ± 1.02 ^Ea^	
Myricetin *O*-methyl ether hexoside deoxyhexoside pentoside ^z^	20	0.97 ± 0.06 ^Aa^	0.31 ± 0.04 ^Bb^	<LOD	<LOD	<LOD	<LOD	0.19
	30	0.81 ± 0.01 ^Ab^	0.41 ± 0.013 ^Ba^	<LOD	<LOD	<LOD	<LOD	
Myricetin *O*-methyl ether hexoside deoxyhexoside ^z^	20	<LOD	0.45 ± 0.05 ^b^	<LOD	<LOD	<LOD	<LOD	0.09
	30	<LOD	0.62 ± 0.05 ^a^	<LOD	<LOD	<LOD	<LOD	
Rutin	20	0.18 ± 0.01 ^Ba^	0.41 ± 0.04 ^Ab^	0.05 ± 0.01 ^Ea^	0.09 ± 0.01 ^Da^	0.15 ± 0.005 ^Ca^	0.09 ± 0.01 ^Da^	0.02
	30	0.20 ± 0.02 ^Ba^	0.54 ± 0.15 ^Aa^	0.08 ± 0.013 ^Ea^	0.10 ± 0.01 ^Da^	0.14 ± 0.013 ^Ca^	0.11 ± 0.01 ^Da^	
Myricetin	20	0.06 ± 0.01 ^Da^	0.85 ± 0.023 ^Da^	18.26 ± 1.65 ^Bb^	21.86 ± 0.75 ^Ab^	4.51 ± 0.37 ^Ca^	3.82 ± 0.11 ^Ca^	0.09
	30	0.07 ± 0.01 ^Da^	0.86 ± 0.03 ^Da^	20.8 ± 0.88 ^Ba^	23.54 ± 0.47 ^Aa^	5.68 ± 0.31 ^Ca^	6.67 ± 0.29 ^Ca^	
Genistein hexoside ^z^	20	1.01 ± 0.09 ^Ca^	1.66 ± 0.39 ^Ca^	6.39 ± 0.49 ^Ba^	7.35 ± 0.28 A ^a^	0.44 ± 0.02 ^Da^	0.54 ± 0.04 ^Da^	0.008
	30	0.98 ± 0.06 ^Ca^	1.09 ± 0.16 ^Ca^	6.61 ± 0.86 ^Aa^	6.81 ± 0.86 A ^a^	1.17 ± 0.03 ^Ba^	1.81 ± 0.05 ^Ba^	
Isorhamnetin 3-*O*-β-glucopyranoside ^z^	20	0.23 ± 0.02 ^Bb^	1.65 ± 0.02 ^Ab^	<LOD	<LOD	<LOD	<LOD	0.01
	30	0.56 ± 0.01 ^Aa^	2.05 ± 0.03 ^Aa^	<LOD	<LOD	<LOD	<LOD	
Quercetin	20	<LOD	<LOD	16.69 ± 0.28 ^Bb^	17.89 ± 1.09 ^Ab^	15.91 ± 0.67 ^Bb^	2.90 ± 0.16 ^Cb^	0.03
	30	<LOD	<LOD	18.39 ± 0.56 ^Ba^	22.00 ± 0.15 ^Aa^	17.63 ± 0.15 ^Ba^	5.22 ± 0.13 ^Ca^	
Biochanin A	20	11.03 ± 0.16 ^Bb^	<LOD	<LOD	12.82 ± 0.35 ^Ab^	<LOD	<LOD	0.01
	30	13.08 ± 0.18 ^Ba^	9.84 ± 0.11 ^C^	<LOD	14.86 ± 0.21 ^Aa^	<LOD	<LOD	
Kaempferol	20	0.25 ± 0.01 ^Db^	0.24 ± 0.01 ^Eb^	1.29 ± 0.02 ^Bb^	3.78 ± 0.03 ^Ab^	1.48 ± 0.01 ^Bb^	0.79 ± 0.02 ^Cb^	0.07
	30	0.63 ± 0.03 ^Da^	0.43 ± 0.01 ^Ea^	2.65 ± 0.05 ^Ba^	6.32 ± 0.19 ^Aa^	2.58 ± 0.05 ^Ba^	1.68 ± 0.01 ^Ca^	
Isorhamnetin	20	<LOD	<LOD	23.19 ± 0.49 ^Bb^	38.06 ± 0.56 ^Ab^	17.05 ± 0.06 ^Ca^	9.48 ± 0.09 ^Db^	0.01
	30	<LOD	0.089 ± 0.01 ^E^	29.24 ± 0.83 ^Ba^	51.18 ± 2.23 ^Aa^	18.11 ± 0.28 ^Ca^	15.28 ± 0.57 ^Da^	

Values expressed in µg/g on a dry weight basis. Mean ± standard deviation (three replicates). ^z^ µg aglycone equivalent/g of sample; DAA, days after anthesis. For the same compound, different capital letters in the same row (A, B, C, D and E) or lowercase letters in the same column (a and b) show significant differences (Fisher test, *p* < 0.05) among genotypes and between developmental stages, respectively. LOD: limit of detection (µg/mL).

## Data Availability

The original contributions presented in this study are included in this article/supplementary material. Further inquiries can be directed to the corresponding author.

## Supplementary Materials

The following supporting information can be downloaded at: https://www.mdpi.com/article/10.3390/plants14162489/s1, Table S1: Phenolic compounds identified by UPLC-ESI-MS in the methanol extracts of developing seeds from chickpea genotypes. Table S2: Gene-specific primers used in RT-PCR.

## Abbreviations

The following abbreviations are used in this manuscript:

UPLC	Ultra-performance liquid chromatography
DAD	Diode array detector
MS	Mass spectrometry
ABTS	2,2′-Azino-bis(3-ethylbenzothiazoline-6-sulfonic acid)
DPPH	1,1-diphenyl-2-picrylhydrazyl
FRAP	Ferric Reducing Antioxidant Power
PAL	Phenylalanine ammonia-lyase
CHS	Chalcone synthase
CHI	Chalcone isomerase
RT-qPCR	Reverse transcription–quantitative polymerase chain reaction
