# Supplementary material for: The Expression of Genes Involved in Phenylpropanoid Biosynthesis Correlates Positively with Phenolic Content and Antioxidant Capacity in Developing Chickpea (Cicer arietinum L.) Seeds
Source: Plants (Basel). 2025 Aug 11;14(16):2489. doi: 10.3390/plants14162489 (PMC12389531; doi:10.3390/plants14162489)
Supplement: Supplementary file 1 [file plants-14-02489-s001.zip › plants-3712922-supplementary.pdf]

Table S1. Phenolic compounds identified by UPLC-ESI-MS in the methanol extracts of developing seeds from chickpea genotypes

Peak	RT (min)	Experimental m/z [M-H]⁻	Theoretical mass (M)	Main fragments	λ_{max} (nm)	Proposed compound	Subclass	Reference
1	1.49	385	386	223 (34.3), 208 (16.5), 191 (3.9), 179 (6.5)	254, 318	Sinapic acid hexoside	Hydroxycinamic acid	[1]
2	1.8	169	170	125 (100), 97 (76.8)	239, 270	Gallic acid[a]	Hydroxybenzoic acid	[1,2]
3	3.04	421	422	289 (30.6), 245 (18.3), 221 (11.3), 203 (8.7), 123 (5.2)	253, 283	Catechin pentoside	Flavan-3-ol	[6]
4	8.11	153	154	137 (90.6), 109 (19.4)	280, 310	Dihydroxybenzoic acid	Hydroxybenzoic acid	[1,3]
5	8.53	289	290	245 (100), 221 (25), 203 (52.3), 151 (10.8), 123 (23.2)	245, 280	Catechin[a]	Flavan-3-ol	[1,2,4,5]
6	10.60	151	152	125 (18.5), 93 (16.3)	280, 315	Vanillin[a]	Hydroxybenzoic acid	[5]
7	12.60	137	138	93 (100)	250	*p*-hydroxybenzoic acid[a]	Hydroxybenzoic acid	[1,3,4]
8	14.62	121	122	77 (100)	282, 332	Benzoic acid	Hydroxybenzoic acid	[1]
9	24.21	163	164	119 (100)	320	*p*-coumaric acid	Hydroxycinamic acid	[1,3]
10	25.99	355	356	193 (69.5), 117 (14.3)	248, 322	Ferulic acid hexoside	Hydroxycinamic acid	[1]

11	28.97	771	772	756 (24.6), 639 (32.9), 331 (39.4), 330 (9.1), 316 (43.94), 315 (76.8), 178 (24.7), 151 (13.3)	245, 357	Myricetin-*O*-methyl ether hexoside deoxyhexoside pentoside	Flavonol	[1]
12	29.20	639	640	331 (52.3), 316 (23.5)	245, 357	Myricetin-*O*-methyl ether hexoside deoxyhexoside	Flavonol	[1]
13	29.53	609	610	301(97.4), 300(40.5), 151 (7.22)	248, 300	Rutin[a]	Flavonol	[3]
14	30.83	317	318	245(16.4), 151(32.5)	250, 348	Myricetin[a]	Flavonol	[5]
15	31.60	431	432	269(34.5), 215(52.6)	250, 320	Genistein hexoside	Isoflavone	[3]
16	31.76	477	478	315 (45.1), 314 (22.3), 300 (12.5), 299 (16.8), 285 (13.4), 271 (7.2)	243, 347	Isorhamnetin 3-*O*-*β*-D-glucopyranoside	Flavonol	[1]
17	33.17	301	302	179 (92.7), 151 (69.5)	253, 369	Quercetin[a]	Flavonol	[6]
18	33.64	283	284	268 (46.0), 253 (2.12), 240 (3.8), 151 (2.55)	260, 322	Biochanin A	Isoflavone	[1,3]
19	35.13	285	286	257(63.0), 229 (20.4), 216 (6.8)	264, 366	Kaempferol[a]	Flavonol	[1]
20	35.57	315	316	301 (13.3), 300 (64.8), 285 (44.6), 261 (9.1)	251, 360	Isorhamnetin[a]	Flavonol	[6]

RT: retention time (min); λmax: maximum absorption wavelength; $[M-H]^-$: molecular ion. [a]Identification of the compound confirmed by the authentic standard.

References

1. Aguilera, Y.; Duenas, M.; Estrella, I.; Hernandez, T.; Benitez, V.; Esteban, R.M.; Martin-Cabrejas, M.A. Phenolic profile and antioxidant capacity of chickpeas (*Cicer arietinum* L.) as affected by a dehydration process. *Plant Foods Hum. Nutr.* **2011**, *66*, 187-195. https://doi.org/10.1007/s11130-011-0230-8
2. Alshikh, N.; de Camargo, A.C.; Shahidi, F. Phenolics of selected lentil cultivars: antioxidant activities and inhibition of low-density lipoprotein and DNA damage. *J. Funct. Foods* **2015**, *18*, 1022-1038. https://doi.org/10.1016/j.jff.2015.05.018
3. Chen, P.X.; Bozzo, G.G.; Freixas-Coutin, J.A.; Marcone, M.F.; Pauls, P.K.; Tang, Y.; Zhang, B.; Liu, R.; Tsao, R. Free and conjugated phenolic compounds and their antioxidant activities in regular and non-darkening cranberry bean (*Phaseolus vulgaris* L.) seed coats. *J. Funct. Foods* **2015**, *18*, 1047-1056. https://doi.org/10.1016/j.jff.2014.10.032
4. Mekky, R.; Contreras, M.d.M.; Elgindi, M.; Abdel-Monem, A.; Abdel-sattar, E.; Segura Carretero, A. Profiling of phenolic and other compounds from Egyptian cultivars of chickpea (*Cicer arietinum* L.) and antioxidant activity: A comparative study. *RSC Adv.* **2015**, *5*, 17751-17767. https://doi.org/10.1039/c4ra13155j
5. Mojica, L.; Meyer, A.; Berhow, M.A.; de Mejía, E.G. Bean cultivars (*Phaseolus vulgaris* L.) have similar high antioxidant capacity, in vitro inhibition of α-amylase and α-glucosidase while diverse phenolic composition and concentration. *Food Res. Int.* **2015**, *69*, 38-48. https://doi.org/10.1016/j.foodres.2014.12.007
6. Quintero-Soto, M.F.; Saracho-Peña, A.G.; Chavez-Ontiveros, J.; Garzon-Tiznado, J.A.; Pineda-Hidalgo, K.V.; Delgado-Vargas, F.; Lopez-Valenzuela, J.A. Phenolic profiles and their contribution to the antioxidant activity of selected chickpea genotypes from Mexico and ICRISAT collections. *Plant Foods Hum. Nutr.* **2018**, *73*, 122-129. https://doi.org/https://doi.org/10.1007/s11130-018-0661-6
